# Effectiveness of Video-Assisted Thoracoscopic Surgery with Bullectomy and Partial Pleurectomy in the Treatment of Primary Spontaneous Pneumothorax—A Retrospective Long-Term Single-Center Analysis

**DOI:** 10.3390/healthcare10030410

**Published:** 2022-02-22

**Authors:** Stephen Fung, Hany Ashmawy, Sami-Alexander Safi, Matthias Schauer, Andreas Krieg, Anja Schauer, Marius Kivilis, Farid Ziayee, Alexander Rehders, Levent Dizdar, Wolfram-Trudo Knoefel

**Affiliations:** 1 Department of Surgery, University Hospital Duesseldorf and Heinrich-Heine-University Duesseldorf, 40225 Duesseldorf, Germany; stephen.fung@med.uni-duesseldorf.de (S.F.); hany.ashmawy@med.uni-duesseldorf.de (H.A.); sami-alexander.safi@med.uni-duesseldorf.de (S.-A.S.); andreas.krieg@med.uni-duesseldorf.de (A.K.); anjamaria.schauer@med.uni-duesseldorf.de (A.S.); marius.kivilis@med.uni-duesseldorf.de (M.K.); rehders@med.uni-duesseldorf.de (A.R.); levent.dizdar@med.uni-duesseldorf.de (L.D.); 2 Department of General and Thoracic Surgery, Augusta Hospital Duesseldorf, 40472 Duesseldorf, Germany; schauer@rocketmail.com; 3 Department of Radiology, University Hospital Duesseldorf and Heinrich-Heine-University Duesseldorf, 40225 Duesseldorf, Germany; farid.ziayee@med.uni-duesseldorf.de

**Keywords:** VATS bullectomy, partial pleurectomy, chest tube, recurrence, PSP

## Abstract

Background: Video-assisted thoracoscopic surgery (VATS) with bullectomy and partial pleurectomy (VBPP) is an increasingly used and well-established surgical treatment for primary spontaneous pneumothorax (PSP). However, reports on its effectiveness and long-term outcomes are limited. The aim of this study was to assess and compare long-term recurrence rates following VBPP and chest tube (CT) treatment and to identify potential risk factors for disease recurrence in patients with PSP. Methods: A total of 116 patients treated either by VBPP or CT were included in this study. Long-term recurrence rates and associations between clinical parameters and recurrence of pneumothorax were analyzed. Results: Sixty-two patients (53.4%) underwent VBPP, whereas 54 (46.6%) patients underwent CT treatment only. During a median follow-up period of 76.5 months, VBPP patients experienced a significantly lower recurrence rate compared to CT patients (6/62 vs. 35/54; *p <* 0.0001). CT treatment (VBPP vs. CT; *p* < 0.001) and a large initial pneumothorax size (Collins < 4 vs. Collins ≥ 4; *p* = 0.018) were independent risk factors for pneumothorax recurrence. Conclusion: VBPP is an effective and safe surgical treatment for PSP. Therefore, patients with a large pneumothorax size might benefit from VBPP, as they are at high risk for disease recurrence.

## 1. Introduction

According to the German S3 guidelines, primary spontaneous pneumothorax (PSP) describes the presence of air without preceding trauma or underlying pulmonary disease within the pleural space of patients under 45 years of age [1]. The incidence of PSP has been reported with approximately 1–9.8 and 7–24 cases per 100,000 individuals per year in females and males, respectively [2,3]. In most cases, PSP results from the rupture of subpleural blebs and bullae, predominantly in young, thin males [4,5]. Despite unknown etiology of PSP, associated risk factors for its occurrence and recurrence, such as male sex, tall stature, nicotine abuse, size of pneumothorax, and a family history of pneumothorax, have been reported [6,7,8,9].

According to the current guidelines, the initial treatment algorithm depending on the patient’s clinical condition includes observation, oxygen supplementation, needle aspiration, and chest tube drainage [1,10,11]. Although PSP often resolves by observational conservative approach or by chest tube drainage [12,13], high rates of recurrence after these treatment modalities have been described [14,15,16,17]. For cases with ipsilateral recurrence of PSP and those with persistent air leak following chest tube (CT) treatment, the guidelines [1,10,11] recommend video-assisted thoracoscopic surgery (VATS). In previous studies, VATS with bullectomy alone was demonstrated to have recurrence rates up to 20% [18,19]. When combined with pleurectomy, the short- and medium-term recurrence rates were reported to reduce to 1–6% [20,21,22]. For long-term results, only a few studies are described in the literature [23,24,25].

Therefore, the primary aim of this study was to evaluate the long-term recurrence rates following VBPP and to compare these results with those of patients successfully treated by CT only at our institution. Secondly, we analyzed underlying clinical features to determine potential risk factors for pneumothorax recurrence in our PSP cohort. 

## 2. Materials and Methods

We retrospectively reviewed data of 120 patients with primary spontaneous pneumothorax (PSP) treated either by VATS bullectomy with partial pleurectomy (VBPP) or by chest tube (CT) only between January 2008 and December 2020 in our institution. Patient demographics, including age, sex, body mass index (BMI), smoking status, length of hospital stay (LOS), duration of surgery, time until recurrence, treatment modality, complications, and size of the pneumothorax, were retrieved from medical records. The size of the pneumothorax was assessed using the regression formula derived from the method of Collins [26]. According to the actual German S3 guidelines for management of spontaneous pneumothorax and post-interventional pneumothorax, a spontaneous pneumothorax (SP) is considered as large when the sum of the interpleural distances derived from Collins method is ≥4 cm [1]. Hence, in this study, we considered a spontaneous pneumothorax to be large at a size of ≥4 cm.

At first presentation of spontaneous pneumothorax (SP), identified patients received chest tube (CT) treatment. Patients who were initially successfully treated with CT during our study period were classified in the CT group. Indication for surgery (VBPP) included persistent air leak for more than 5 days following CT treatment (*n* = 20), first ipsilateral recurrent pneumothorax (recurrence of pneumothorax on the previously treated side, *n* = 32), synchronous bilateral spontaneous pneumothorax (*n* = 8), and spontaneous hemopneumothorax (*n* = 2). Of note, patients who received VBPP for recurrence either underwent initial CT treatment at our institution before our study period or had received CT treatment at another hospital and subsequently presented in our institution with recurrence. Prior to surgery, a computer tomography of the chest was performed to detect any bullous disease. A team of three specialized thoracic surgeons (WTK, AS, AR) made indication for surgery. Patients with incomplete follow-up data and patients who received other treatment modalities (e.g., thoracotomy, apical pleurectomy (pleurectomy of the apex of the pleural cavity only), observation, needle aspiration) were excluded from this study. The primary objectives of this study were to assess and compare long-term recurrence rates after treatment with VBPP and CT in our institution and to identify potential risk factors for pneumothorax recurrence. Recurrence was described as an ipsilateral pneumothorax detected on a chest radiograph at presentation in our emergency room after surgical treatment by VBPP or chest tube drainage. The local ethic committee of the Heinrich-Heine University Clinic of Duesseldorf approved this study (study no.: 2020-1271).

### 2.1. Surgical Technique: VATS Bullectomy with Partial Pleurectomy (VBPP)

Our specialized team of thoracic surgeons (A.S., A.R., and W.T.K.) performed all surgical procedures and postoperative patient follow-up. All the patients were treated under general anesthesia with a double-lumen tube intubation and single-lung ventilation. After lateral positioning of the patient, video-assisted thoracoscopic surgery (VATS) was performed in the conventional two- or three-port approach. Initial thoracoscopy was undertaken for thorough inspection of the visceral and parietal pleura. Bullectomy was carried out when blebs or bullae were identified by wedge resection using an endoscopic stapling device (Autosuture GIA Universal; COVIDIEN^TM^, Mansfield, MA, USA). Partial pleurectomy was performed beginning from the apex of the pleural cavity. During this procedure, the parietal pleura was carefully separated from the endothoracic fascia while sparing the region of the subclavian artery and vein to avoid injury of these structures. Pleurectomy was performed up to the 7th or 8th intercostal space in a blunt manner. After cautious hemostasis of the endothoracic fascia using electrocautery to reduce the risk of hemothorax, one 24-Fr. chest tube was inserted and connected to a digital underwater seal system (Thopaz+, Medela AG, Baar, Switzerland) with a suction of −20 mm Hg. During postoperative care, the chest tube drain was removed when no clinical signs of air leak and a drain output of less than 200 mL after 24 h were evident. After chest tube removal, a chest radiograph was taken to verify full expansion of the lung. All the patients (VBPP and CT treated patients) received our standard postoperative medication regime of analgesia (non-opioid, orally or intravenously). The patients received either metamizole-natrium 1000 mg, paracetamol 1000 mg, or ibuprofen 600 mg four times per day. In cases of persistent pain using the standard pain medication regime, we applied piritramide (opioid) 7.5 mg intravenously every 4–6 h on patient request.

### 2.2. Outpatient Care and Follow-Up

One week after discharge, the patients visited our outpatient clinic for postoperative control and follow-up. These visits continued at a 3-month interval for one year. A chest radiograph was taken at each visit. The patients were advised to visit our emergency room at any time they had symptoms related to recurrent pneumothorax, such as dyspnea, chest pain, or cough. Recurrent pneumothorax was identified clinically in each case with a chest radiograph. For patients who recurred after CT or VBPP treatment, VBPP or re-VATS was performed, respectively. For long-term follow-up, patients were contacted and assessed with a questionnaire.

### 2.3. Statistical Analysis

All data were analyzed with the SPSS 25.0 software program (Statistical Package for Social Sciences; SPSS Inc., Chicago, IL, USA). Patients’ data were expressed as numbers, mean or median. Continuous variables were compared using a Mann–Whitney U test, and the chi-square test was implemented for categorical data. Recurrence-free survival (RFS) was defined as the period between initial treatment by surgery (VBPP) or chest tube (CT) and ipsilateral recurrent pneumothorax. Kaplan–Meier curves were generated and evaluated with the log-rank test (Mantel–Cox test), and hazard ratios (HRs) were estimated with 95% confidence intervals (CIs). For multivariate analysis, all variables were included in a logistic regression analysis. Statistical significance was considered at *p* < 0.05.

## 3. Results

Between January 2008 and December 2020, 120 patients with primary spontaneous pneumothorax (PSP) were treated either by VATS-bullectomy with partial pleurectomy (VBPP) or by chest tube (CT) in our institution. Four patients were lost during follow-up and were excluded from this analysis. A total of 116 patients with a median age of 24 years (range 18–41) were included in this study. Sixty-two patients underwent VBPP, whereas 54 patients received CT treatment. The clinical characteristics of the patients are summarized in Table 1.

At presentation, the mean pneumothorax sizes estimated according to Collins method were 13.4 cm and 13.9 cm in the VBPP and CT groups, respectively, indicating a large pneumothorax size for both patient cohorts. Clinical variables, such as age, gender, BMI, and pneumothorax size and chest tube duration, were similar in both groups (Table 1). Three patients suffered a hemothorax in the VBPP group. Of these patients, two were successfully treated conservatively, while one patient underwent re-VATS. Likewise, 24 patients suffered a prolonged air leak following VBPP. In all the cases, the patients were successfully treated conservatively. Significant differences were found between the two groups in terms of patients’ smoking behaviour and the length of hospital stay (LOS). The proportion of smokers was significantly higher and the LOS significantly longer in the VBPP group compared to the CT group. However, it must be considered that the LOS in the VBPP group was significantly prolonged by the preoperative period until surgery was performed (mean of 4.1 days). 

During a median follow-up period of 76.5 months (range 1–155 months), patients who underwent CT treatment experienced a significantly higher recurrence rate compared with patients following VBPP (VBPP vs. CT: 9.7% vs. 64.8%; *p* < 0.0001). This high rate of recurrence in the CT group occurred mainly within the first year after treatment (CT vs. VBPP: 9.6 months (range 2–26) vs. 59 months (range 6–110); *p* < 0.001). Interestingly, patients with a large pneumothorax size (Collins ≥ 4 cm) suffered a significantly higher rate of recurrence compared with patients with a small pneumothorax size (Collins < 4 cm vs. Collins ≥ 4 cm: 9.1% vs. 37%; *p* = 0.010) (Table 2).

**Figure 1:**
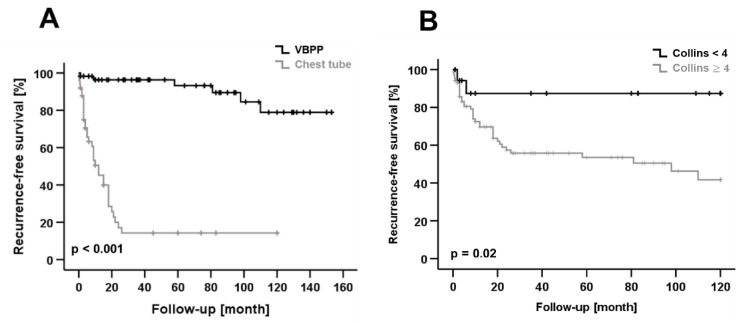
(**A**) Kaplan–Meier curve shows recurrence-free survival (RFS) after treatment by surgery (VBPP) or chest tube (CT). VBPP was associated with significantly better RFS compared to CT treatment. (**B**) Patients with a large pneumothorax size (Collins ≥ 4 cm) had a significantly reduced RFS compared to patients with a small pneumothorax size (Collins < 4 cm).

Next, we investigated potential risk factors for recurrent pneumothorax in our patient cohort. Univariate analysis revealed that treatment of PSP patients with VBPP (VBPP vs. CT: HR 0.056; CI: 0.023–0.14; *p* < 0.001) and a small size of the initial pneumothorax (Collins ≥ 4 cm vs. Collins < 4 cm: HR 4.602; CI: 1.106–19.151; *p* = 0.020) were significantly associated with a lower risk of pneumothorax recurrence (Table 3; Figure 1A,B). Both factors, namely the therapeutic procedure chosen (VBPP vs. CT: HR 0.047; CI: 0.017–0.132; *p* < 0.001) and the baseline pneumothorax size (Collins ≥ 4 cm vs. Collins < 4 cm: HR 6.325; CI: 1.372–29.162; *p* = 0.018), were confirmed as independent predictive markers of pneumothorax recurrence in multivariate regression analysis (Table 4).

## 4. Discussion

Nowadays, VATS–bullectomy with partial pleurectomy (VBPP) is a well-established and increasingly used surgical treatment for primary spontaneous pneumothorax (PSP). Recently, VBPP has been reported with superior performance and low rates of recurrence compared to VATS bullectomy alone [20,21,24,25]. However, only a few studies elucidate its effectiveness in terms of long-term outcomes. Additionally, studies that examine potential risk factors for PSP recurrence following surgical treatment are rare. In this study, we evaluated the long-term recurrence rates of patients treated with VBPP or CT in our institution. Moreover, we examined underlying clinical factors that might influence disease recurrence in our patient cohort.

In our study, 62 patients with a mean age of 24.6 years underwent VBPP, while 54 patients (mean age 25.3 years) (Table 1) were successfully treated by chest tube. Compared to some previous studies in the literature with a high number of smokers in their PSP collective [12,16,17], we had a significantly low number of smokers in both groups of our patient cohort (Table 1). This might be due to the increased awareness campaigns and information about the negative effects of smoking on human health over the last years. Interestingly, the length of hospital stay (LOS) of patients in the VBPP group was significantly longer than in the CT group. Although the LOS in the VBPP group was consistent with previous studies reporting the hospitalization time after VATS in the literature [27,28], this result was significantly prolonged by the number of hospital days until surgery was performed (Table 1). 

During a follow-up period of 76.5 months (range 1–155 months), six (9.7%) patients suffered a recurrence following VBPP. Compared to the VBPP group, the CT group experienced a higher recurrence rate of 64.8% (35 patients recurred, Table 2). This difference was statistically significant in the univariate (VBPP vs. CT: HR 0.056; CI: 0.023–0.14; *p* < 0.001) and multivariate analyses (VBPP vs. CT: HR 0.047; CI: 0.017–0.132; *p* < 0.001) (Table 3 and Table 4). These results demonstrate that VBPP is associated with an increased recurrence-free survival compared to CT treatment. The recurrence rate of 9.7% of our VBPP cohort is slightly higher than some studies described in the literature. In the study of Imperatori, A. et al. [23] with 134 patients, VATS blebectomy and parietal pleurectomy had a recurrence rate of 6%. The median follow-up was 79 months (range: 36–187 months). Shaikhrezai, K et al. [24] observed no recurrent pneumothorax of 44 PSP patients operated by VATS bullectomy and partial pleurectomy during a median follow-up of 73 months. In a recent study of Caecilia Ng et al. [25], in which 73 PSP patients underwent VATS partial pleurectomy, a recurrence rate of 1.4% at a median follow-up period of 58.5 months was reported. Although the above recurrence rates are lower than the recurrence rate of our VBPP cohort, none of them evaluated the pneumothorax size as a potential risk factor for PSP recurrence. In our study, the patients presented with a large pneumothorax size estimated according to the method of Collins. The mean pneumothorax size of the VBPP group was 13.4 cm and 13.9 cm for the CT group. As reported in earlier studies, a large pneumothorax size is a risk factor for PSP recurrence [9,29,30]. Therefore, we assumed that the large pneumothorax size of our PSP cohort influenced our comparatively high rate of recurrence.

To determine potential risk factors for recurrence, we analyzed the impact of clinical factors, such as gender, age, BMI, smoking status, pneumothorax size, and the treatment modality on PSP recurrence. Treatment by CT placement (VBPP vs. CT: HR 0.047; CI: 0.017–0.132; *p* < 0.001) and a large pneumothorax size (Collins ≥ 4 cm vs. Collins < 4 cm: HR 6.325; CI: 1.372–29.162; *p* = 0.018) proved to be independent risk factors for disease recurrence in our PSP patient cohort. These results highlight the high efficacy of VBPP in the treatment of patients with PSP and the clinical importance of the initial pneumothorax size as a significant risk factor for disease recurrence. Noteworthy, other potential risk factors for disease recurrence, such as family history of pneumothorax, presence of connective tissue disorders, cannabis consumption, and scoliosis, were also investigated. However, the small number of patients with these risk factors limited the statistical analysis, and thus, they were not included as risk factors in this study.

The indication to perform surgery (VBPP) might differ internationally. However, our data demonstrate that the risk of recurrent pneumothorax can be significantly reduced by VBPP. In addition, our data highlight the fact that a large initial pneumothorax size is associated with a markedly increased risk of pneumothorax recurrence. Obviously, the power of our study is limited due to its retrospective design and the small number of patients included, but the logical consequence based on our observations would be to prefer VBPP to simple chest drainage in patients with a large pneumothorax size. However, this observation should be verified in large prospective randomized trials.

## 5. Conclusions

Our data confirm that VBPP is an effective and safe surgical treatment for PSP. It is associated with a very low risk of disease recurrence. A large pneumothorax size is an independent risk factor for recurrence of PSP. Therefore, the option of VBPP should be discussed early for PSP patients treated with a large pneumothorax size, and close follow-ups should be performed due to the high risk of recurrence.

## Figures and Tables

**Table 1:** Clinical characteristics of the VBPP and CT group.

Variables	VBPP *n* = 62 (%)	CT *n* = 54 (%)	*p-*Value
**Gender**			
Male	48 (77.4)	38 (70.4)	
Female	14 (22.6)	16 (29.6)	0.376
**Smoking status**			
Smokers (current and past)	19 (30.6)	8 (14.8)	
Non-smokers	43 (69.4)	46 (85.2)	0.028 *
**Pneumothorax size (cm)**			
Initial size at presentation (mean)	13.4	13.9	0.778
Collins < 4 cm (n)	13 (21.0)	9 (16.7)	
Collins ≥ 4 cm (n)	47 (75.8)	42 (77.8)	
Missing (n)	2 (3.2)	3 (5.5)	0.527
**Age (y)**			
**Median (Mean)**	23 (24.6)	24.(25.3)	0.537
**Height (m)**			
Median (Mean)	1.8 (1.8)	1.8 (1.8)	1.000
**Weight (kg)**			
Median (Mean)	64.5 (67.1)	70 (68.6)	0.332
**BMI (kg/m^2^)**			
Median (Mean)	20.5 (20.7)	21.4 (21.0)	0.348
**Length of hospital stay (LOS) (days)**			
Mean (range)	6.1 (3−13)	4.8 (2−7)	<0.001 *
**Days until operation**			
Mean (range)	4.1 (0−11)	/	/
**Operation time (min)**			
Mean (range)	79.4 (45–130)	/	/
**Chest tube duration (days)**			
Mean (range)	5.5 (3−8)	5.2 (2−6)	0.836
**Time until recurrence (months)**			
Mean (range)	59 (6−110)	9.6 (2−26)	<0.001 *
**Complications (n)**			
Hemothorax	3 (4.8)	0 (0.0%)	0.103
Prolonged air leak after surgery	24 (38.7)	/	/

Data are presented as mean, median, numbers (*n*) and percentages, BMI, body mass index; kg, kilogram; LOS, length of hospital stay; CT, chest tube; min, minutes; VBPP, VATS bullectomy with partial pleurectomy; m, meter; cm, centimeter; y, years. * *p*-value < 0.05 indicates statistical significance.

**Table 2:** Patient clinical characteristics and recurrence rates of PSP.

Variable	Recurrence *n* (%)	*p*-Value
**Gender**		
Male	29 (33.7)	0.323
Female	12 (40.0)	
**Age**		
≤24 years	22 (33.4)	0.756
>24 years	19 (36.5)	
**BMI**		
≤20.85 kg/m^2^	20 (35.5)	0.843
>20.85 kg/m^2^	21 (36.2)	
**Smoking status**		
Smokers (current and past)	6 (22.2)	0.186
Non-smokers	35 (39.3)	
**Treatment**		
VBPP	6 (9.7)	<0.0001 *
CT	35 (64.8)	
**Pneumothorax size (cm)**		
Collins < 4 cm	2 (9.1)	0.010 *
Collins ≥ 4 cm	37 (41.6)	

Kg, kilogram; m, meter, VBPP, VATS bullectomy with partial pleurectomy; CT, chest tube; BMI, body mass index. * *p*-value < 0.05 indicates statistical significance.

**Table 3:** Univariate analysis of potential risk factors for recurrence of PSP.

Risk Factor	Hazard Ratio	95% CI	*p*-Value
**Gender**			
Male vs. female	0.864	0.44–1.695	0.668
**Age**			
>median vs. ≤median	1.088	0.588–2.011	0.787
**BMI**			
>median vs. ≤median	1.155	0.623–2.139	0.644
**Smoking status**			
Smoker vs. non-smokers	0.536	0.220–1.246	0.133
**Treatment**			
VBPP vs. CT	0.056	0.023–0.14	<0.001 *
**Pneumothorax size**			
Collins ≥ 4 vs. Collins < 4	4.602	1.106–19.151	0.020 *

Univariate analysis displays potential risk factors that might influence recurrence-free survival (RFS). Patients with a large pneumothorax size (Collin ≥ 4) and those treated by chest tube (CT) have a significantly low RFS. * *p*-value < 0.05 indicates statistical significance. VBPP, VATS bullectomy with partial pleurectomy; BMI, body mass index; CI, confidential interval.

**Table 4:** Multivariate analysis of potential risk factors for recurrence of PSP.

Risk Factor	Hazard Ratio	95% CI	*p*-Value
**Treatment**			
VBPP vs. CT	0.047	0.017–0.132	<0.001 *
**Pneumothorax size**			
Collins ≥ 4 vs. Collins < 4	6.325	1.372–29.162	0.018 *

Multivariate analysis displayed initial pneumothorax size and administered treatment as independent risk factors for PSP recurrence. Chest tube (CT) treatment and a large pneumothorax size (Collins ≥ 4) were associated with a significantly reduced recurrence-free survival. * *p*-value < 0.05 indicates statistical significance. VBPP, VATS bullectomy with partial pleurectomy; CI, confidential interval.

## Data Availability

The data presented are included in this study; the corresponding author on request may provide additional data.
